# A pragmatic cluster randomised controlled trial of telehealth on disease specific quality of life in patients’ with chronic obstructive pulmonary disease and their health-related quality of life and psychological distress over 1 year in the Whole System Demonstrator programme

**Published:** 2012-06-15

**Authors:** Lorna Rixon, Shashivadan P Hirani, Martin Cartwright, Michelle Beynon, Helen Doll, Stanton P Newman, WSD Research Team

**Affiliations:** City University, UK; City University, UK; City University, UK; City University, UK; University of East Anglia, UK; City University, UK; City University, UK

**Keywords:** telehealth, COPD, Quality of life, Whole System Demonstrator

## Abstract

**Introduction:**

There is limited evidence for the effectiveness of TH on quality of life (QoL) in patients with COPD. A systematic review in the area inclusive of all respiratory conditions indicated that there were no UK based studies, or randomised controlled trials (RCTs) evaluating the effectiveness of TH for COPD (Janna et al. 2009). A more recent systematic review found 6 studies, only two of which measured QoL as an outcome (Bolton et al. 2010). One of these studies was a RCT and found improvements in QoL at 3 months (Koff 2009), while the other was a non-controlled before and after study which found no difference in quality of life scores at 6 months (Trappenburg, 2008). Research in this area is plagued by small sample sizes, absence of longer-term follow-ups, insufficient descriptions of the intervention, poor internal validity of whether using the device in the context of a complex healthcare intervention leads to improved outcomes for the patient, and few attempts to measure quality of life in patients with COPD following the introduction of these devices.

**Aims and objectives:**

The current investigation is part of the Whole System Demonstrator (WSD) programme that aims to evaluate the effectiveness of telehealth (TH) for patient reported outcomes with Chronic Obstructive Pulmonary Disease (COPD). The primary objective was to evaluate the effectiveness of telehealth for COPD specific QoL and to examine whether there were improvements in HRQoL and psychological distress at short-term and long-term follow-up in this cohort of patients with COPD.

**Methods:**

WSD is one of the largest pragmatic cluster randomised controlled trials evaluating TH in the UK. Patients with COPD from three regions in England (Cornwall, Kent and Newham) were recruited from 179 GP practices randomised balancing for region, practice size, deprivation index, non-white proportion and prevalence of COPD. Over 570 patients with COPD completed a comprehensive battery of questionnaires assessing a range of patient reported outcomes. Measures of generic Health-Related Quality of Life (HRQoL) (Short Form-12), disease specific QoL including perceived control over COPD, fatigue caused by the disease and the emotional impact of the disease (Chronic Respiratory Questionnaire). Psychological distress was measured by depression (CESD-10) and anxiety (STAI).

**Results:**

Multi-level modelling was utilised to evaluate the effect of trial arm on HRQoL and COPD specific QoL. Results for intention-to-treat analysis, participants that received the intervention as per the research protocol, complete case analysis for cases with all baseline, short-term and longer-term follow-ups completed and an available case analysis of patients with either a short or long-term follow-up available. The results will be discussed and have important clinical implications for COPD management.

**Conclusions:**

Results and conclusions are censored until any findings are published in peer-reviewed journals.

